# Carbon Dots-Decorated Bi_2_WO_6_ in an Inverse Opal Film as a Photoanode for Photoelectrochemical Solar Energy Conversion under Visible-Light Irradiation

**DOI:** 10.3390/ma12101713

**Published:** 2019-05-27

**Authors:** Dongxiang Luo, Qizan Chen, Ying Qiu, Baiquan Liu, Menglong Zhang

**Affiliations:** 1 School of Materials and Energy, Guangdong University of Technology, Guangzhou 510006, China; luodx@gdut.edu.cn (D.L.); 18219435079@163.com (Q.C.); 2 Guangdong Research and Design Center for Technological Economy, Guangzhou 510000, China; srawoyjs@sina.com; 3 Luminous! Center of Excellence for Semiconductor Lighting and Displays, School of Electrical and Electronic Engineering, Nanyang Technological University, Singapore 639798, Singapore; bqliu@ntu.edu.sg

**Keywords:** photoelectrode, carbon dots, macroporous electrode

## Abstract

This work focuses on the crystal size dependence of photoactive materials and light absorption enhancement of the addition of carbon dots (CDs). mac-FTO (macroporous fluorine-doped tin oxide) films with an inverse opal structure are exploited to supply enhanced load sites and to induce morphology control for the embedded photoactive materials. The Bi_2_WO_6_@mac-FTO photoelectrode is prepared directly inside a mac-FTO film using a simple in situ synthesis method, and the application of CDs to the Bi_2_WO_6_@mac-FTO is achieved through an impregnation assembly for the manipulation of light absorption. The surface morphology, chemical composition, light absorption characteristics and photocurrent density of the photoelectrode are analyzed in detail by scanning electron microscopy (SEM), transmission electron microscopy (TEM), X-ray diffraction (XRD), UV–vis diffuse reflectance spectra (DRS), Energy dispersive X-ray analysis (EDX) and linear sweep voltammetry (LSV).

## 1. Introduction

The energy crisis is one of the major social problems that various countries will encounter in the 21st century [1,2]. Semiconductor photocatalysis is regarded as a potential green technology for mitigating the energy crisis [3,4,5]. Fujishima and Honda used TiO_2_ as a photocatalyst to split water to produce oxygen and hydrogen under ultraviolet light radiation [6]. Photocatalytic water splitting is a good strategy for converting solar energy to chemical energy. As a device for solar energy, the performance of photoanodes is reliant on their light absorption, charge carrier separation and catalysis/electrolyte diffusion [7].

Many efforts have been made to improve photoelectrochemical (PEC) water splitting efficiency using various modifications such as ion doping [2], heterostructure [6] and loading co-catalysts [8], assemblies which have proved to be beneficial in suppressing the charge carrier recombination in photocatalytic materials. For instance, composites including Pt/TiO_2_ [8,9], BiOBr/Bi_2_WO_6_ [10], g-C_3_N_4_/KTaO_3_ [11], Pt/ZnO [12], CdS/ZnS [13] and Ag/CdS [14] have been synthesized and have exhibited enhanced photocatalytic activity. Photoactive materials with nanostructures, including nanofibers [15], nanosheets [16] and nanotubes [17,18], have also been developed to improve photocatalytic activity. 

Among the photoactive materials, transition metal sulfides (such as CdS [19,20], Znln_2_S_4_ [21,22] and iron group elements like Fe-, Ni- and Co-based sulfides [23,24,25,26,27]) with a relatively narrow bandgap are sensitive to most of the visible wavelength region. However, the photogenerated holes tend to oxidize the catalyst itself, rather than water, in the absence of sacrificial reagent, resulting in photocorrosion. On the other hand, metal oxide semiconductors typically have high chemical stability, while the bandgap energies of metal oxides are commonly higher than those of sulfides because the O (2p) orbital exhibits a lower energy than the S (2p) orbital. Among the metal oxides, Bi_2_WO_6_ has received extensive attention due to its moderate bandgap (2.7–2.8 eV) (Figure S3), chemical stability and non-toxicity [28,29,30,31]. However, Bi_2_WO_6_ suffers from an unsatisfactory photo-response range and rapid recombination of the photogenerated carriers. The photocatalytic activity and surface reaction of semiconductor photocatalysts are highly dependent on the band structures, light photoresponse range and the specific surface area of the catalysts [32,33,34]. Sensitizing photocatalysts with dyes or good light absorbers is one of the strategies employed to enhance the utilization of solar energy [3,35,36,37,38,39,40]. To this end, carbon materials including carbon dots [41], carbon nanotubes [42] and grapheme [43,44] were exploited in order to extend the absorption range or to optimize the charge separation. 

Recently, carbon dots (CDs) have been employed in photocatalysis systems due to their excellent photophysical and chemical properties, such as ease of synthesis, non-toxicity and low cost [45,46,47]. In addition, CDs can serve as co-catalysts to enhance the light harvesting capacity and accumulate charge separation [48,49] in photocatalytic systems. In this work, Bi_2_WO_6_ is directly synthesized into macroporous fluorine-doped tin oxide (mac-FTO) films (Bi_2_WO_6_@mac-FTO) and the CDs are subsequently decorated on Bi_2_WO_6_@mac-FTO photoanodes. The optical, morphological and photocatalytic properties of these samples are investigated to evaluate the impact of CDs on a mac-FTO-based photoanode.

## 2. Experimental

### 2.1. Reagents and Materials

Bismuth nitrate pentahydrate (Bi(NO_3_)_3_·5H_2_O, 99%), ethylenediamine (C_2_H_8_N_2_, 99%), ethanol (CH_3_CH_2_OH, 99.7%), nitric acid (HNO_3_, 68%), sodium sulfate decahydrate (Na_2_SO_4_·10H_2_O, 99%), sodium sulfite anhydrous (Na_2_SO_3_, 98%), citric acid monohydrate (C_6_H_8_O_7_·H_2_O, 99.8%), sodium tungstate dihydrate (Na_2_WO_4_·2H_2_O, 99.5%), hydrogen peroxide (H_2_O_2_, 30 v%), sulfuric acid (H_2_SO_4_, ≥95%), crimp headspace vials (c2183-01-100EA), planar FTO glass (p-FTO) (11 Ω/sq) and monodispersed polystyrene spheres (d = 450 nm, 2.5 wt%) were purchased from Aladdin industrial Co., Ltd. (Shanghai, China) and used as received. The deionized water used throughout all experiments was purified through a Millipore system (Millipore, Billerica, MA, USA). 

### 2.2. Synthesis of the Polystyrene Film Template

The polystyrene film template was prepared by a simple evaporation method. The conductive surface of clean p-FTO glass was coated with polystyrene monodisperse pellets by the surface tension of the solution. The p-FTO slide (2 × 10 × 15 mm) was first immersed in a piranha solution (H_2_SO_4_:H_2_O_2_ = 3:1, volume ratio) for 2 h, then washed with deionized water and dried under N_2_. The clean p-FTO glass was placed vertically in a crimp headspace vial (10 mL) containing a suspension of polystyrene monodisperse spheres (PS:ethanol = 3:80, volume ratio) dispersed in 100% ethanol. The suspension was just higher than the top of the p-FTO slide. The obtained glass vials were then transferred to a muffle furnace and kept at 58 °C for 15 h until the volatiles were completely evaporated. Finally, the electrode was removed and a polystyrene film template was obtained.

### 2.3. Fabrication of the Mac-FTO Electrode

The macroporous fluorine-doped tin oxide (mac-FTO) film with 3D porous space structures was synthesized using a facile thermal polymerization method. First, 1.4 g of SnCl_4_·5H_2_O (4 mmol) was dissolved in 20 mL of ethanol before being sonicated for 2 min. Then, to obtain the mac-FTO precursor solution, 0.24 mL of saturated NH_4_F solution (2 mmol) was added dropwise into the above solution and the mixture was sonicated for 10 min. The PS film template was pre-soaked in ethanol for 0.5 h and then transferred and soaked in the mac-FTO precursor solution for 1 h. The PS film template was then removed and placed in a crucible and sintered in the air atmosphere at 450 °C for 2 h at 1 °C/min, and was finally left to naturally cool to room temperature. The resulting mac-FTO film provided more attachment sites for Bi_2_WO_6_ catalysts. 

### 2.4. Synthesis of the Bi_2_WO_6_@mac-FTO Photoelectrode

The Bi_2_WO_6_@mac-FTO photoelectrode was prepared using an in situ synthesis method. Bi(NO_3_)_3_·5H_2_O (1 mmol) was dissolved in 30 mL of diluted HNO_3_ (pH = 3) and Na_2_WO_4_·2H_2_O (0.5 mmol) was dissolved in 20 mL of deionized water. Both solutions were vigorously stirred until clear. The mac-FTO slides were immersed in a Bi(NO_3_)_3_ solution for 1 min then transferred and immersed in Na_2_WO_4_ solution for 1 min. The mac-FTO slides were immersed alternately in the Bi(NO_3_)_3_ and Na_2_WO_4_ solutions with 20, 60 or 100 times to obtain photoelectrode with different thicknesses. The electrodes were then calcined in an air atmosphere at 600 °C for 2 h at 3 °C·min^−1^, and finally left to naturally cool to room temperature. The obtained Bi_2_WO_6_@mac-FTO electrodes were heat-treated at 720 °C for 2 min.

### 2.5. Synthesis of the Bi_2_WO_6_@mac-FTO Photoelectrode Decorated with CDs

CDs were synthesized via a hydrothermal method. First, 1.47 g of C_6_H_8_O_7_·H_2_O was dissolved in 14 mL of deionized water, and 0.47 mL of C_2_H_8_N_2_ was added dropwise into the above solution. The mixture was then sonicated for 10 min. The obtained solution was then transferred to an autoclave and kept at 200 °C for 5 h in a muffle furnace, before finally being allowed to naturally cool to room temperature. To obtain the CD solution, the solution obtained from this reaction was subjected to dialysis using a dialysis bag under vigorous magnetic stirring, which the molecular weight cutoff (MWCO) of the dialysis bag is 1000 dalton. The Bi_2_WO_6_@mac-FTO photoelectrode was then immersed in CD solution for 2 h. Finally, the Bi_2_WO_6_@mac-FTO photoelectrode was removed from the CD solution and dried in a vacuum oven at 60 °C for 24 h to obtain the photoelectrode, which the CDs decorated on the Bi_2_WO_6_@mac-FTO photoelectrode (CDs/Bi_2_WO_6_@mac-FTO).

## 3. Sample Characterization

The morphology and structure of the as-prepared samples were investigated using a Hitachi SU8220 field emission scanning electron microscope (Hitachi High Co., Ltd., Japan at different amplifications and an accelerating voltage of 15 kV. Low-resolution transmission electron microscopy images and high-resolution transmission electron microscopy images were obtained using an FEI Talos F200S transmission electron microscope (FEI Co., Ltd., USA at an accelerating voltage of 200 KV, and elemental mapping of the as-prepared electrodes was conducted. Powder from the as-prepared electrodes was scraped off and dissolved in ethanol before characterization and the suspension was dispersed by ultrasound. A drop of this suspension was added into a 3 mm diameter micro-grid copper film. The TEM sample was obtained after the drying treatment. XRD patterns were recorded using a Bruker D8 ADVANCE diffractometer (Bruker Co., Ltd., Germany. The crystal structure and composition were measured with CuKa (λ = 0.15406 nm) radiation (40 KV, 30 mA). The datas of UV–vis diffuse-reflectance spectra were collected on an ultraviolet–visible diffuse reflectance spectrometer and a clean p-FTO glass was used as a reflectance standard.

Photoelectrochemical measurements were made using a standard three-electrode setup. A platinum sheet (10 × 10 mm) was used as the counter electrode and the reference electrode was Ag/AgCl (3 M KCl internal solution). Connection to the as-prepared samples working electrode was achieved using copper tape and the bottom 10 mm of the electrode was immersed in the electrolyte solution. The electrolyte was 0.5 M Na_2_SO_4_ (pH = 7). A xenon lamp was used as solar light simulator and the light intensity was adjusted to 100 mW·cm^−2^. Potentials were referenced to the reversible hydrogen electrode standard using the following formula [50]:

(1) $$E_{\mathrm{RHE}}=E_{\mathrm{vs}.\left(\mathrm{Ag}/\mathrm{AgCl}\right)}+E_{\mathrm{ref}\left(\mathrm{Ag}/\mathrm{AgCl}\right)}+0.059\mathrm{PH}$$

where E_ref(Ag/AgCl)_ = 0.209 V vs. NHE at 25 °C.

To study the electrochemical kinetics at the interface between the electrode and the electrolyte, a three-electrode system was adopted for electrochemical impedance spectroscopy (EIS) (Ametek Co., Ltd., Berwyn, PA, UK). The physical-electrochemical properties of the Bi_2_WO_6_@mac-FTO photoelectrode with applied CDs, the Bi_2_WO_6_@mac-FTO photoelectrode and the unmodified Bi_2_WO_6_@mac-FTO photoelectrode were examined in a 0.1 M NaSO_4_ electrolyte solution under light and dark conditions. The real factor (Z’) and imaginary factor (Z”) of characteristic Nyquist plots were used to calculate the charge transfer resistance (Rct) between the electrode and electrolyte interfaces, solution resistance (Rs) and diffusion coefficient. A small range of an EIS semicircle corresponds to a low Rct value and a higher electrical conductivity.

## 4. Results and Discussion

### 4.1. Structural Characterization

**Figure 1:**
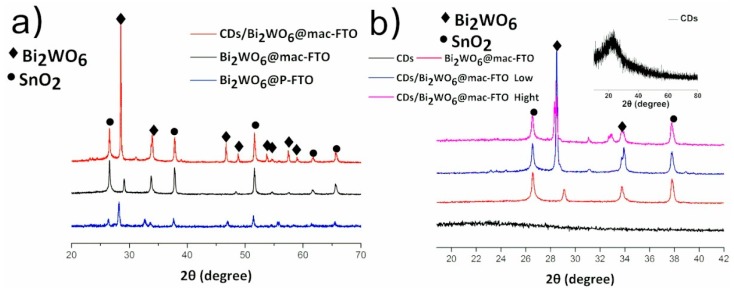
(**a**) XRD in which ◆ = JCPDS 73-2020 for Bi_2_WO_6_, ● = JCPDS 46-1088 for SnO_2_; (**b**) enlargement of (a) from 2Ɵ = 20 to 42°, insert image is the XRD pattern of carbon dots (CDs), JCPDS 50-0926. The low and high labels indicate Bi_2_WO_6_@mac-FTOs decorated with low and high amounts of CDs, respectively.

The XRD patterns of Bi_2_WO_6_, SnO_2_ and CDs can be identified as shown in Figure 1, in which the diffraction patterns are consistent with JCPDS (joint committee on powder diffraction standards) 73-2020, 46-1088 and 50-0926, respectively. Direct evidence of Bi_2_WO_6_ can be confirmed from the diffraction peaks obtained for the Bi_2_WO_6_@p-FTO. The diffraction peaks at 2θ = 26.57°, 37.76°, 51.77°, 61.74° and 65.74° in the XRD pattern of the Bi_2_WO_6_@mac-FTO and CDs/Bi_2_WO_6_@mac-FTO photoelectrodes correspond to the (110), (200), (211), (310) and (301) lattice planes of SnO_2_. No obvious diffraction peaks from CDs could be detected after CDs were introduced, presumably due to their low population on the surface of the CDs/Bi_2_WO_6_@mac-FTO photoelectrode. Figure 1b shows an XRD image of CDs which has a broad peak centered at 22.76°, indicating the presence of CDs [45]. An enlarged pattern of the diffraction peaks in the range of 2θ = 20–42° is shown in Figure 1b, which suggests that with an increase in CD content on the surface of the Bi_2_WO_6_@mac-FTO photoelectrode, the peak position is shifted slightly towards a lower 2θ value, indicating that CDs have been successfully doped into the Bi_2_WO_6_ nanomaterial [51].

**Figure 2:**
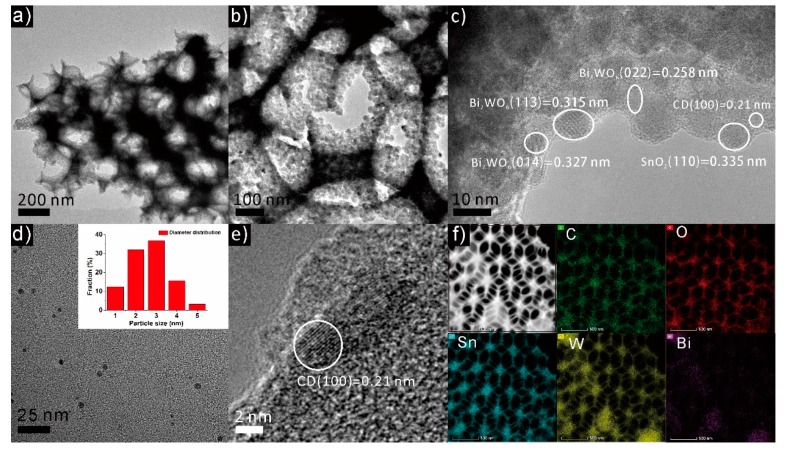
(**a**–**c**) TEM images of the CDs/Bi_2_WO_6_@mac-FTO photoelectrode. (**d**) TEM images of the CDs. (**e**) HR-TEM and (**f**) TEM-EDX elemental mapping of the CDs/Bi_2_WO_6_@mac-FTO photoelectrode.

The texture and structure and of the as-prepared samples were characterized by TEM. Figure 2a,b show the LR-TEM images of the CDs/Bi_2_WO_6_@mac-FTO photoelectrodes. In Figure 2c, the HR-TEM image displays well-resolved lattice fringes with interplanar distances of 0.327 nm, 0.315 nm and 0.258 nm indexed to the (014), (113) and (022) lattice planes of Bi_2_WO_6_, respectively. The lattice fringe with 0.335 nm spacing can be assigned to the (110) lattice plane of SnO_2_. As shown in Figure 2d, the TEM image of the CDs suggests that the synthesized CDs are nearly spherical and have an average size of approximately 2.5 nm (Figure 2e). The TEM images and corresponding elemental mapping images (Figure 2f) of the CDs/Bi_2_WO_6_@mac-FTO photoelectrode indicate that the C, O, W, Sn and Bi elements are distributed uniformly on the as-prepared sample. The above results further confirm that the CDs were successfully decorated on surface of the Bi_2_WO_6_@mac-FTO photoelectrode.

**Figure 3:**
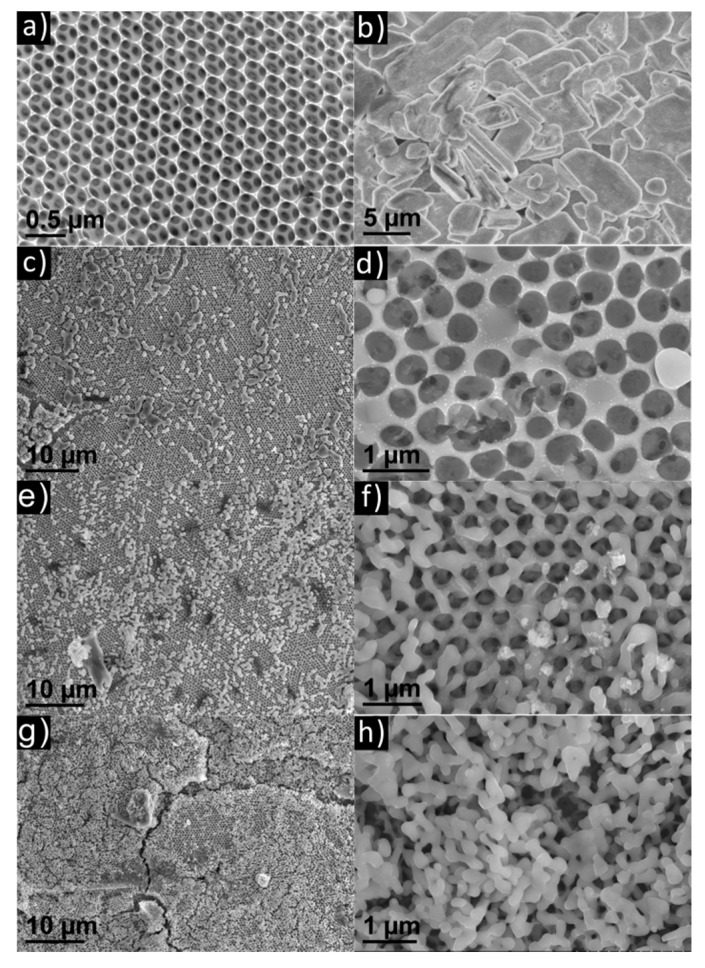
(**a**) SEM image of mac-FTO film. (**b**) SEM image of the Bi_2_WO_6_@p-FTO photoelectrode displays a stacked lamellar morphology. (**c**–**h**) SEM images of CDs/Bi_2_WO_6_@mac-FTO photoelectrodes (the cycles are 20, 60 and 100, respectively), and the corresponding SEM images showing the small size filling of the skeleton.

SEM was employed to investigate the texture, structure and morphology of the as-prepared samples, and revealed that the mac-FTO film on the p-FTO substrate exhibits a long-range ordered porous structure (Figure 3a), and SEM image of cross section of mac-SnO_2_ electrode (Figure S2). As a control, the Bi_2_WO_6_ synthesized on p-FTO displays the typical stacked lamellar structure (ca. 6 µm) of pure Bi_2_WO_6_ (Figure 3b). On the other hand, the as-prepared Bi_2_WO_6_@mac-FTO (Figure 3c–h) shows a reduced size (ca. 100 nm) of Bi_2_WO_6_ due to the crystal size restraint effect from the sub-micro porous substrate. This phenomenon has also been observed in an α-Fe_2_O_3_@mac-SnO_2_ system [7]. A reduced size of photoactive material typically indicates a shorter pathway for charge migration, which allows faster transfer of photogenerated carriers. In addition, as the cycle coefficient increases, the porous structure of the mac-FTO film is blocked.

### 4.2. Optical Properties

**Figure 4:**
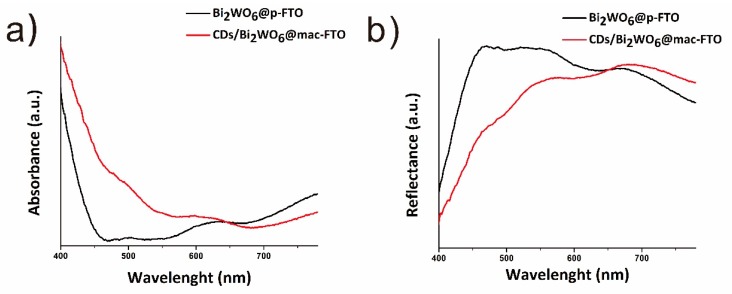
(**a**) Optical absorption and (**b**) reflection spectra of the Bi_2_WO_6_@p-FTO and CDs/Bi_2_WO_6_@mac-FTO photoelectrodes.

UV–vis transmittance was employed to compare the light absorptivity of samples. As shown in Figure 4, enhanced light absorbance was observed in the CDs/Bi_2_WO_6_@mac-FTO photoelectrode under wavelengths shorter than 660 nm. This enhancement can be attributed to the addition of CDs and suggests a more efficient utilization of solar energy. As shown in Figure S1, digital photographys of CDs solution which exposed to visible light and 250 nm UV light, and the PL spectra of CDs under different excitation wavelength.

### 4.3. Photoelectrochemistry

**Figure 5:**
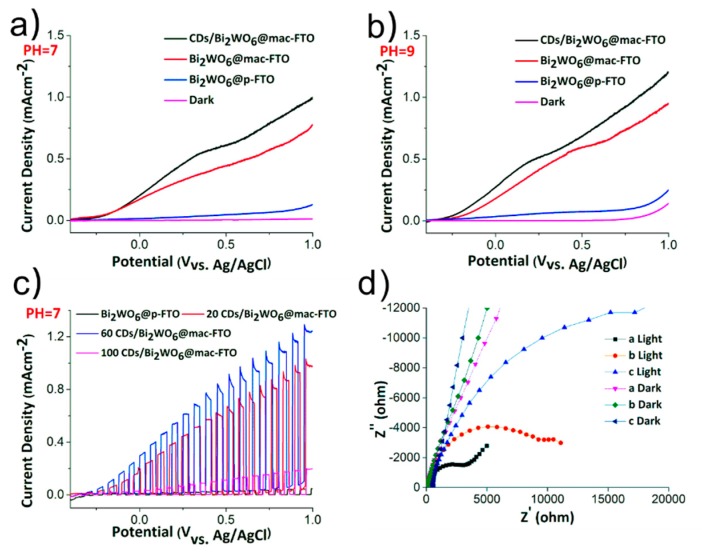
(**a**,**b**) Linear sweep voltammetry (LSV) curves of the Bi_2_WO_6_@p-FTO photoelectrode, Bi_2_WO_6_@mac-FTO photoelectrode and CDs/Bi_2_WO_6_@mac-FTO photoelectrode (the experiments are conducted in electrolytic solutions with pH = 7 and pH = 9, respectively). (**c**) Linear sweep voltammogram of the CDs/Bi_2_WO_6_@mac-FTO photoelectrodes, wherein the mac-SnO_2_ film was immersed in precursor solution 20, 60 and 100 cycles (**d**) Electrochemical impedance spectroscopy (EIS) Nyquist plots of the photoelectrodes. The labels of a, b and c indicate the CDs/Bi_2_WO_6_@mac-FTO, Bi_2_WO_6_@mac-FTO and Bi_2_WO_6_@p-FTO photoelectrodes, respectively. The light and dark labels indicate the conditions of the test.

Linear sweep voltammetry (LSV) experiments were conducted under chopped illumination to estimate the photoelectrochemical performance of the as-prepared samples. The Bi_2_WO_6_@mac-FTO photoelectrode exhibited significantly improved photoactivity in comparison to the Bi_2_WO_6_@p-FTO photoelectrode and previous reports [52]. To be more specific, the CDs/Bi_2_WO_6_@mac-FTO, Bi_2_WO_6_@mac-FTO and Bi_2_WO_6_@p-FTO photoelectrodes had photocurrent densities of 0.202, 0.171 and 0.014 mA·cm^−2^, at 0 V vs. V_Ag/AgCl_, respectively, (Figure 5a). This enhancement can be attributed to the high surface area and good light absorption supplied by the mac-FTO substrate and CDs, respectively. Furthermore, the attachment of CDs is pH- and ionic strength-dependent. The pH dependence of the as-prepared samples was investigated (pH = 5 to 10) and the highest photocurrent density (0.272 mA·cm^−2^) of the CDs/Bi_2_WO_6_@mac-FTO photoelectrode was obtained at pH = 9 (0 V vs. V_Ag/AgCl_) (Figure 5b). This can be attributed to the accumulation of charge migration by surface hydroxyl groups [6]. In addition, the dependence of the photoactivity of the Bi_2_WO_6_@mac-FTO on the amount of modified CDs is shown in Figure 5c, revealing that CDs/Bi_2_WO_6_@mac-FTO photoelectrodes with 60 soaking cycles exhibited a significant photoresponse. The addition of CDs could indeed optimize the light absorption both in the UV and visible regions, as has been seen previously in CQDs/Bi_5_O_7_I [51], CQDs/TiO_2_, CQDs/Bi_2_O_3_ [53] and CQDs/TNTs (TiO_2_ nanotubs) [54] composites. This could be because the photon absorption capability of CDs in the samples leads to more efficient PEC/photocatalytic performance. However, the excessive loading of CDs will reduce the surface area of Bi_2_WO_6_ exposure to electrolyte. Next, EIS was exploited to study the electrochemical kinetics at the interfaces between the electrode and electrolyte. The range of the EIS semicircle of the electrodes was smaller under illumination than under dark conditions. The diameter of the arc radius on the EIS Nyquist plot of the Bi_2_WO_6_@mac-FTO photoelectrode with applied CDs was smallest, suggesting a smaller interface resistance for the CDs/Bi_2_WO_6_@mac-FTO photoelectrode. The low resistance of the CDs/Bi_2_WO_6_@mac-FTO photoelectrode could be attributed to the presence of CDs in the composite. When CDs are immobilized on the Bi_2_WO_6_@mac-FTO photoelectrode, the electron transfer resistance (Ret) decreased considerably because of the perfect electrical conductivity of CDs, and they are responsible for the higher electrical conductivity. Photogenerated electrons could be transferred to the surface of the electrode faster through the CDs. In consideration of the stability of CD-modified Bi_2_WO_6_, although the addition of CDs can improve the photoelectrochemical performance, relatively worse stability was observed in comparison to pristine Bi_2_WO_6_. As shown in Figure S4, we carried out a 20 cycle PEC test (each cycle of the photoelectrodes lasted 5 min under illumination at 0 V vs. V_Ag_/_AgCl_). The CD-modified electrode had a larger current density, but the current decreased by about 22% after 20 cycles, as compared to a 13% reduction in the pristine Bi_2_WO_6_ photoelectrode. This could be due to the detachment of CDs from Bi_2_WO_6_. Bi_2_WO_6_ exhibited a yellow color, and after the application of CDs a dark brown sample was obtained. Subsequently, the dark brown sample turned to light brown after 20 cycles, suggesting that the CDs had detached. However, the CD-modified sample had a photocurrent density that was 20% greater than that of the control group. In addition, it should be noted that, although the carboxylic group on CDs would improve the CDs anchoring on the surface of metal oxides [55], the attachment is pH- and ionic strength-dependent. As shown in Figure S5, we came up with a possible mechanism for the enhanced photocatalytic activity of the CD/Bi_2_WO_6_@mac-FTO photoelectrode.

## 5. Conclusions

Bi_2_WO_6_@mac-FTO photoelectrodes were successfully synthesized through an in situ synthesis method and then decorated with CDs. The obtained photocurrent density of the CDs/Bi_2_WO_6_@mac-FTO photoelectrode was higher than that of the initial Bi_2_WO_6_@p-FTO photoelectrode, substantiating the superiority of the CDs/Bi_2_WO_6_@mac-FTO photoelectrode. This superiority was manifested in the light absorption and large surface area. Mace-FTO film with a 3D porous structure was applied in order to create a larger surface area and to control the growth of the Bi_2_WO_6_ catalyst. In contrast to a p-FTO film, crystals of Bi_2_WO_6_ can have smaller particle sizes. In addition, the CDs applied to the Bi_2_WO_6_@mac-FTO photoelectrode exhibited an optimized photocurrent density of up to 0.202 mA·cm^−2^ under light at 0 V vs. V_Ag/AgCl_ and 1 mA·cm^−2^ at 1 V vs. V_Ag/AgCl_ (pH = 7). The improved photocurrent density generation of the CDs/Bi_2_WO_6_@mac-FTO photoelectrode can be attributed to the suitable morphology control from mac-FTO films and the application of CDs to the photoelectrode. The application of CDs enhances the light absorption intensity and expands the photoresponse under visible light irradiation, which gives some insight for similar solar energy conversion experiments. 

## Supplementary Materials

The following are available online at https://www.mdpi.com/1996-1944/12/10/1713/s1, Figure S1. Digital photographs of CDs solution while exposed to visible light and 250 nm UV light, and the PL spectra of CDs under different excitation wavelengths; Figure S2. SEM image of the cross-section of a mac-SnO_2_ electrode; Figure S3. The image of the bandgap of pure Bi_2_WO_6_ photocatalyst; Figure S4. Linear sweep voltammogram of Bi_2_WO_6_@mac-FTO and CDs/Bi_2_WO_6_@mac-FTO photoelectrodes at 0 V vs. VAg/AgCl (pH = 7); Figure S5. Schematic illustration of the possible mechanism for the enhanced photocatalytic activity of the CDs/Bi2WO6@mac-FTO photoelectrode.
