# Supplementary material for: Carbon Dots-Decorated Bi2WO6 in an Inverse Opal Film as a Photoanode for Photoelectrochemical Solar Energy Conversion under Visible-Light Irradiation
Source: Materials (Basel). 2019 May 27;12(10):1713. doi: 10.3390/ma12101713 (PMC6566223; doi:10.3390/ma12101713)
Supplement: Supplementary file 1 [file materials-12-01713-s001.pdf]

materials

MDPI

Supporting Information

Carbon Dots-Decorated Bi_2WO_6 in an Inverse Opal Film as a Photoanode for Photoelectrochemical Solar Energy Conversion under Visible-Light Irradiation

Dongxiang Luo [1], Qizan Chen [1], Ying Qiu [2], Baiquan Liu [3] and Menglong Zhang [1,*]

[1] School of Materials and Energy, Guangdong University of Technology, Guangzhou 510006, China; luodx@gdut.edu.cn (D.L.); 18219435079@163.com (Q.C.)
[2] Guangdong R&D Center for Technological Economy, Guangzhou 510000, China; srawoyjs@sina.com
[3] Luminous! Center of Excellence for Semiconductor Lighting and Displays, School of Electrical and Electronic Engineering, Nanyang Technological University, Singapore 639798, Singapore; bqliu@ntu.edu.sg
* Correspondence: mlzhang@m.scnu.edu.cn

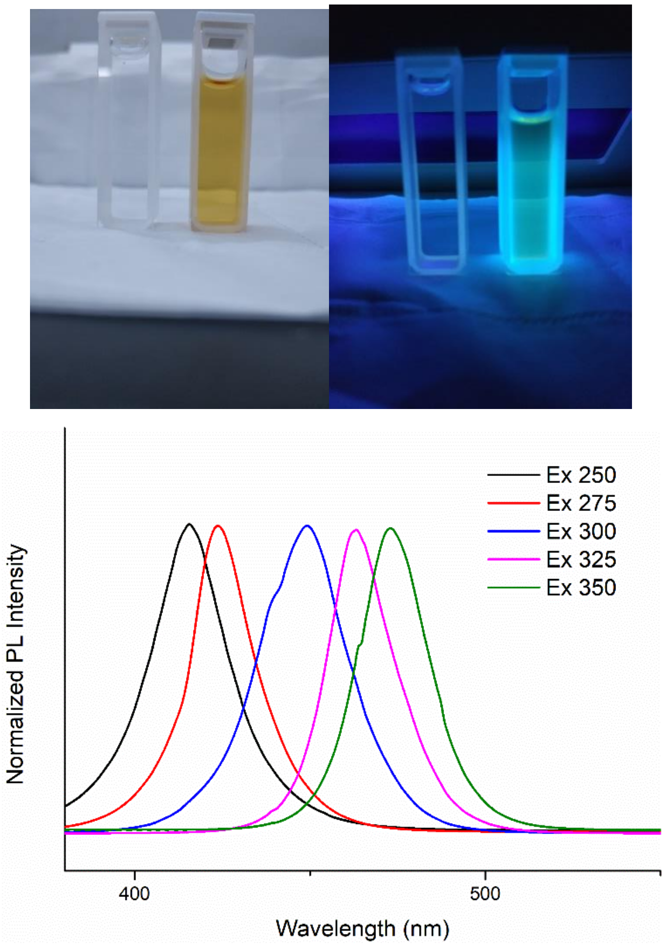

Figure S1. Digital photographs of CDs solution while exposed to visible light and 250 nm UV light, and the PL spectra of CDs under different excitation wavelengths.

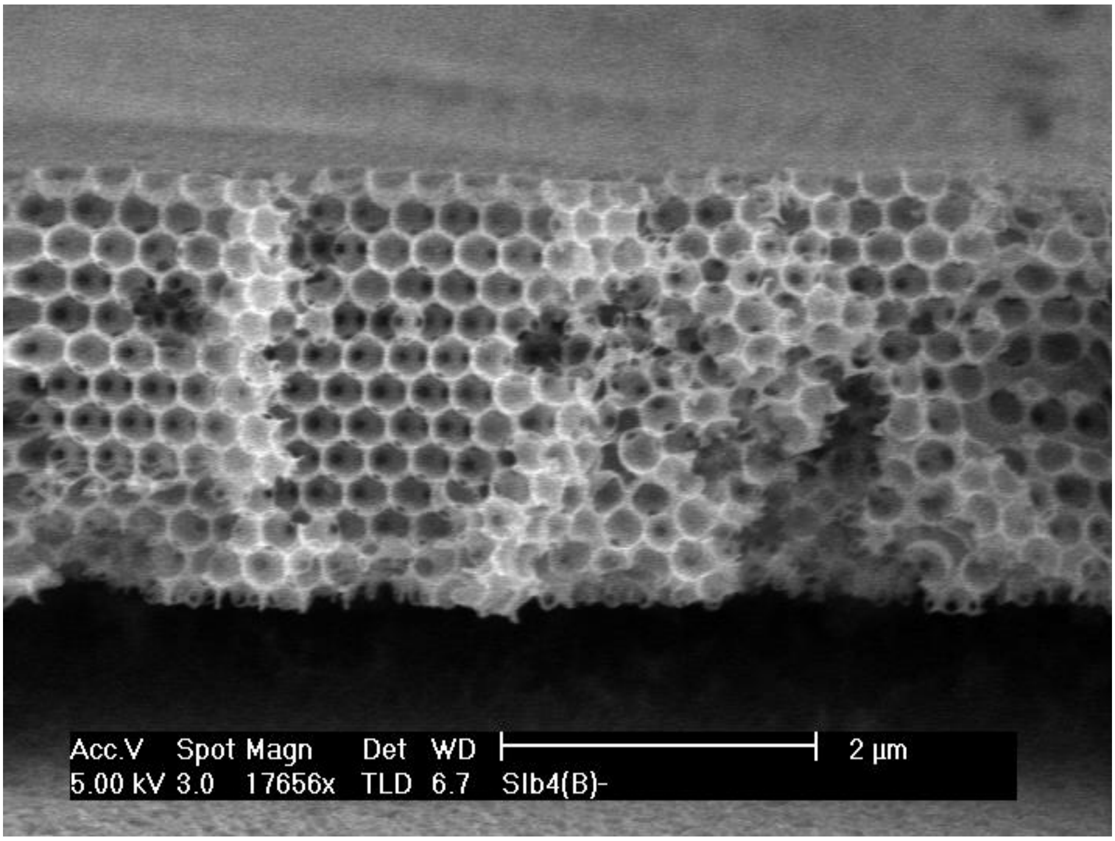

Figure S2. SEM image of the cross-section of a mac-SnO_2 electrode.

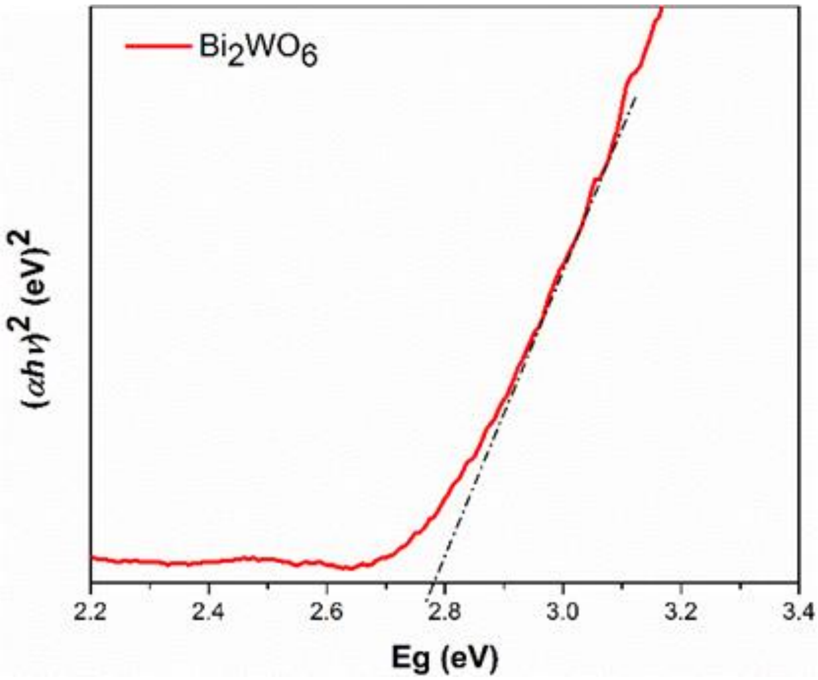

Figure S3. The image of the bandgap of pure Bi_2WO_6 photocatalyst.

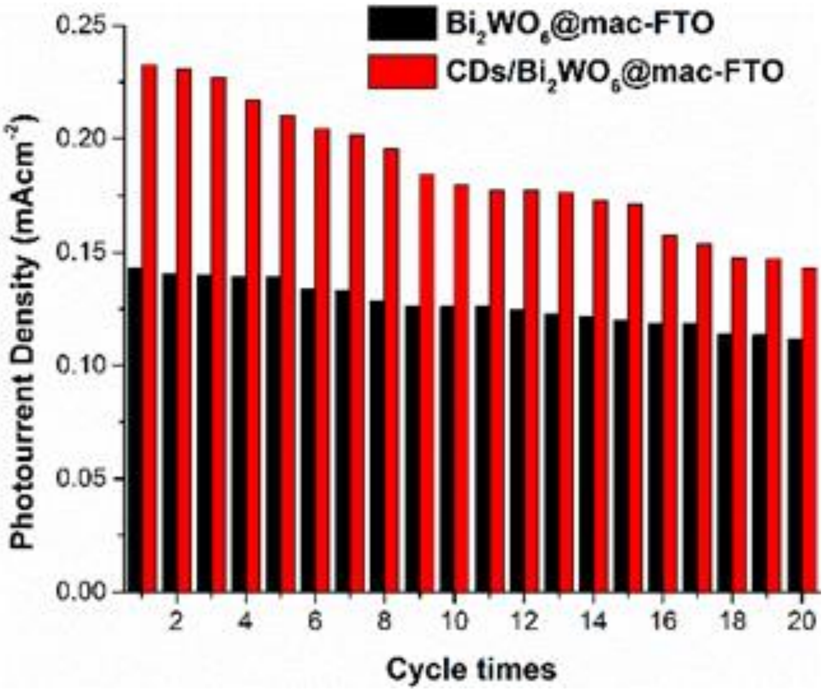

Figure S4. Linear sweep voltammogram of Bi_2WO_6@mac-FTO and CDs/Bi_2WO_6@mac-FTO photoelectrodes at 0 V vs. $V_{Ag/AgCl}$ (pH = 7).

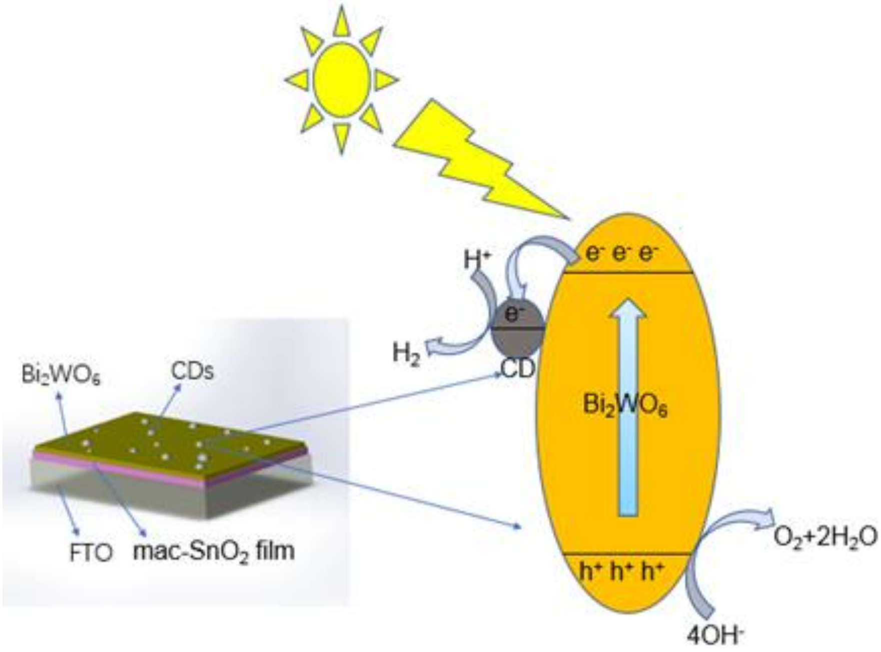

Figure S5. Schematic illustration of the possible mechanism for the enhanced photocatalytic activity of the CDs/Bi_2WO_6@mac-FTO photoelectrode.
